# Case of Small-pox Occurring a Third Time after Vaccination, and Proving Fatal

**Published:** 1851-07

**Authors:** John Webster


### RECORD OF MEDICAL SCIENCE.

### PATHOLOGY AND PRACTICE OF MEDICINE.

*Case of Small-pox occurring a third time after Vaccination, and proving Fatal.* By JOHN WEBSTER, M. D., F. R. S.—Three well-marked examples of the recurrence of small-pox, met with in the same family, were related, one of which terminated fatally. The case especially referred by Dr. Webster was that of H. N. N., who had been vaccinated satisfactorily in 1827, when three months old. Notwithstanding this circumstance, he was attacked by small-pox in 1833, along with an elder brother, who had been likewise vaccinated. Both patients recovered. In 1838 the two lads were again attacked by variola, along with a third brother, likewise regularly vaccinated. All three got quite well in due time. Subsequently, Mr. H. N. N. went to India in the Company's service, where he was seized, in April, 1850, with the usual and well-marked symptoms of small-pox, which soon became confluent, and proved fatal at Dharwarinth, on the 13th of that month; this making the third time this gentleman had been attacked by variola, although previously vaccinated.—*Lon. Journ. of Med., May,* 1851.

